# Inhibitory control mediates the association between body mass index and math performance in children: A cross-sectional study

**DOI:** 10.1371/journal.pone.0296635

**Published:** 2024-04-11

**Authors:** Felipe Barradas Cordeiro, Marcos Guilherme Moura-Silva, Mauro Roberto de Souza Domingues, Mizael Carvalho de Souza, Renan Rocha, Irene Esteban-Cornejo, Natáli Valim Oliver Bento-Torres, Kirk I. Erickson, João Bento-Torres

**Affiliations:** 1 Graduate Program in Human Movement Science, Neurodegeneration and Infection Research Laboratory, João de Barros Barreto University Hospital, Federal University of Pará, Belém, Brazil; 2 Institute of Mathematics and Scientific Education, Federal University of Pará, Belém, Brazil; 3 Department of Physical Education and Sports, Faculty of Sport Sciences, Sport and Health University Research Institute (iMUDS), University of Granada, Granada, Spain; 4 CIBER de Fisiopatología de la Obesidad y Nutrición, Instituto de Salud Carlos III, Madrid, Spain; 5 AdventHealth Research Institute, Orlando, Florida, United States of America; Universidad Nacional Autonoma de Mexico, MEXICO

## Abstract

**Background:**

Overweight and obesity affect more than 18% of children and adolescents in the world. Obesity-related associations with brain morphology might be associated with reduced efficiency of inhibitory control. This association highlights a possible mechanism by which obesity impacts intelligence and academic achievement. Prior work indicates a mediating effect of inhibitory control on the relationship between body mass index (BMI) and intelligence and academic achievement. However, although obesity is associated with impaired math performance, we do not know whether inhibitory control also mediates the relationship between BMI and math performance. This study tests the hypothesis that inhibitory control statistically mediates the relationship between BMI and math performance.

**Methods:**

161 children (9 to 13 years old, 80 female) participated in the present study. We evaluated BMI; math performance, in a test composed of 20 arithmetic equations of the type *x* = (*a* × *b*) − *c*; and inhibitory control through the Flanker test. We carried out Spearman correlation tests, hierarchical multiple linear regression, and tested the confidence of the model where inhibitory control statistically mediates the indirect association between BMI and math performance. Mediation analysis in this cross-sectional study aimed to improve understanding of indirect relationships and offer insights into possible causal connections.

**Results:**

Better math performance and lower BMI were associated with greater accuracy on the inhibitory control test and greater accuracy on the inhibitory control test was associated with better performance on math test. We found an indirect association between higher BMI in children and impairments in math performance, that was mediated by inhibitory control (a: -0.008, p = 0.025; b: 7.10, p = 0.0004; c: 0.05, p = 0.592; c’: 0.11, p = 0.238; Indirect Effect: -0.0599, 95% CI: -0.13, -0.005).

**Conclusions:**

An indirect association between higher body mass indices in children and impairments in math performance was detected, through the impact that BMI has on inhibitory control.

## Introduction

Overweight and obesity affect more than 1.9 billion adults worldwide and high body mass index (BMI) alone was responsible for 4.7 million deaths in 2017 [1, 2]. The prevalence of these disorders among children and adolescents is higher than in adults and is growing, reaching more than 18% of the population in this age group [2]. More than half of children with obesity will be adolescents with obesity and are five times more likely to become adults with obesity compared to children without obesity [3].

Being overweight and obese are associated not only with the development of several pathologies [4], but also with worse school performance (e.g., math performance) [5–7], impaired development of the prefrontal cortex, and deficits in executive functions [8] potentially associated with a reduced P3 anteriorization and diminished N2 amplitude during an inhibitory task [9]. Particularly, children with obesity have deficits in inhibitory control [10], that is, in their ability to selectively attend to certain information while ignoring irrelevant information. Inhibitory control is a predictor of better academic performance in children [11], while faster reaction times on attention tasks are related to math skills in preschool children, showing that difficulties in numerical skills may be related to low levels of attention or slowness in performing tasks [12].

Inhibition control plays a critical role in regulating the activation of concurrent representations during the retrieval of mathematical facts [13]. A recent meta-analysis examined the relationship between executive functions and academic achievement throughout the elementary school years. The findings of this analysis revealed that inhibitory control emerges as the executive function most strongly correlated with math fluency [13]. Indeed, more recently, a study demonstrated how inhibitory control could forecast suboptimal mathematical achievement in students across grade levels 2, 6, and 10 [12]. Previous research also has shown that inhibitory control is essential for suppressing irrelevant and conflicting responses during mental calculations [14]. For instance, when solving addition operations such as 3 + 2, it is necessary to inhibit responses associated with different operations, like multiplication (e.g., 3 × 2), especially when these operations have been rote memorized. Additionally, inhibitory control plays a crucial role in preventing operational errors, such as the erroneous substitution of mathematical operations (e.g., addition for multiplication) during the retrieval of arithmetic facts stored in long-term memory [15]. Therefore, the ability to inhibit inappropriate responses and maintain the selection of the appropriate mathematical operation emerges as an essential cognitive skill for the precise execution of mathematical tasks and the effective retrieval of arithmetic facts [16].

Inhibitory control is a significant statistical mediator in elucidating the link between Body Mass Index (BMI) and non-specific academic performance [17]. Furthermore, it is worth noting that inhibitory control exhibits a discernible correlation with counterintuitive reasoning during adolescence, thereby substantiating the conjecture that inhibitory control plays a pivotal role in the domains of scientific education and mathematical reasoning [18]. It is of significance to underscore that underperformance in mathematics can potentially impact a child’s self-concept concerning their mathematical prowess, consequently engendering negative emotions and diminishing the likelihood of them pursuing careers in science, technology, engineering, and mathematics fields [19].

One plausible physiological explanation pertains to children aged 6 to 8 years old with overweight or obese, who manifest diminished executive function capabilities and exhibit a smaller right hippocampus in comparison to their normal-weight counterparts [20]. Moreover, robust evidence demonstrates a negative association between higher BMI and thinner prefrontal cortex in two large-scale cross-sectional studies involving children aged 8 to 11 years. Additionally, cortical thickness statistically mediates the relationship between BMI and executive function [8, 21]. Indeed, there exists an association between smaller hippocampi and diminished inhibitory control, while a higher cortical volume correlates with enhanced mathematical achievement in overweight or obese children in the 8–11 age range [22]. Genetic factors also are implicated in the BMI and Executive function association by accounting for around 80% of the phenotypes [23]. The performance on academic and cognitive tasks is negatively related to visceral adipose tissue in obese kids, but in normal-weight ones, this relationship was positive, suggesting that the association between adiposity and cognition could be dependent on the IMC status [24].

Inhibitory control functions as a mediating factor in the influence of BMI on overall academic achievement, as assessed by a composite score encompassing nine subjects. This mediation accounts for approximately 36% of the total effect observed in adolescents aged 13.5 to 17 years [17]. Similarly, in adults with overweight and obesity, individual variations in inhibitory control statistically mediate the relationship between BMI and cognitive intelligence. These behavioral associations are consonant with the neurobiological alterations mentioned earlier in prefrontal regions [25]. However, as much as obesity impairs math performance [5–7], we still do not know whether inhibitory control statistically mediates the relationship between BMI and arithmetic performance in early adolescents.

Therefore, this study aims to investigate the relationship between body mass index (BMI) and mathematical performance in children, considering the role of inhibitory control. To achieve this goal, the following specific tasks will be performed: Examine the relationship between BMI and inhibitory control with math performance in children; Investigate whether the possible association between inhibitory control and mathematical performance differs in terms of BMI categories; Test whether inhibitory control mediates the relationship between BMI and mathematical performance in children.

Our first hypothesis is that there is a negative relationship between body mass index (BMI) with inhibitory control and arithmetic skills and a positive relationship between inhibitory control and mathematical performance in children. Given that these cognitive variables may exhibit specific correlations contingent upon the BMI range, we also believe that the association between inhibitory control and mathematical performance may be different in children with different BMI categories. Lastly, and most importantly, we believe that inhibitory control plays a mediating role in the relationship between BMI and mathematical performance in children.

## Method

### Study overview and participants

A total of 255 children were initially considered eligible for this study. Of these, 174 children were examined for eligibility and 161 were confirmed eligible. All 161 eligible children (9 to 13 years old, 80 female) participated in the study and completed the follow-up period, and their data were included in the analysis.

Fifth and sixth-grade students from two Brazilian elementary schools participated in the present study. For School 1, the recruitment period extended from February to March 2018, and data collection took place from March to August 2018. For School 2, recruitment occurred from July to August 2019, and data collection was conducted from September to December 2019.

The inclusion criteria for this study are children between 9 and 13 years of age who do not have any mathematical learning disorders, progressive blindness, deafness, or chronic heart or neurological disease. Based on similar studies, it was expected that the effect sizes (Cohen’s d) of this research would fall within the range of 0.10 and 0.24 [17, 25]. To ensure that our sample size of 161 was sufficient for detecting these effect sizes, a post-hoc power analysis was conducted using GPower 3.1.9.7 [26]. The results revealed that our sample size had sufficient power between 0.96 and 0.99 when using an alpha of 0.05. Furthermore, a sensitivity power analysis indicated that our sample could detect effect sizes larger than 0.05 with an alpha of 0.05 and a power of 0.8.

The assessment sessions took place during class hours in which the children usually study, in a schoolroom reserved for the study procedures, and under controlled temperature and lighting conditions and noise minimization. Our procedures were conducted in accordance with the protocol published on the Protocols.io platform, which provides a detailed description of the methods and procedures used in this study [27]. Teste All participants were naive about the purposes of the study. Initially, weight and height measurements were performed, followed by five minutes of rest in a sitting position. Participants were asked to sit on a chair, positioned 50 cm away from a computer screen, which displayed a white background. After the rest period, participants performed the math performance test, and cognitive performance assessment (inhibitory control). The experimental session lasted an average of 25 minutes. The legal guardians of each child completed an anamnesis questionnaire informing the history of diagnosed pathologies, medication use, routine habits, and the child’s diet. The Research Ethics Committee approved this study (CAAE: 76887417.2.0000.0018).

### Specific procedures

#### Math performance test

The math performance test was displayed on a monitor (1366x768 pixels) with a gray background and composed by arithmetic equations of the type: *x* = (*a* × *b*) − *c*, being necessarily: *a* ≠ *b* and *c* > 0 [28]. For instance, participants were presented with equations such as "*x* = 4 × 2 − 5", "*x* = 8 × 3 − 2", and "*x* = 5 × 2 − 1". The children were instructed to perform the mental calculation and answer aloud for recording by the researcher. The use of fingers was allowed as an aid to calculate the correct answer.

The test execution was limited up to 10 minutes, although the participants were not aware of this condition. Participants were instructed to solve as many questions as accurately as possible. Since performance effectiveness is often measured by accuracy [29], we adopted this metric to assess arithmetic performance in the current study.

#### Inhibitory control assessment

The Flanker test is a non-verbal test used to assess inhibitory control. Participants were instructed to respond as quickly and accurately as possible indicating the direction of the central arrow (target), and to ignore adjacent arrows that could be congruent (same direction) or incongruent (opposite directions) to the target. If the central arrow is pointed to the right, the keyboard “Shift-right” button should be pressed, and if the central arrow is pointed to the left, the “Shift-left” button on the keyboard should be pressed.

The test was semi-automated (Psychology Experiment Building Language software—PEBL) and displayed on a screen monitor (1366x768 pixels), located 50 cm in front of the child, initially showing a black background. The stimuli (white arrows with a size equivalent to 100 pixels) were displayed randomly for a maximum time of 800 ms or until the participant responded. The interval between stimuli was 1000 ms, configured for 9 initial adaptation stimuli with immediate feedback and 200 evaluation stimuli for the analysis. After the initial adaptation, the child was asked if they understood the task. If their response was negative, a new adaptation simulation was performed. The accuracy of responses to incongruent trials was used as the index of inhibitory control.

#### Classification by body adiposity index–BMI

BMI is an index used by the World Health Organization, including for children [30]. BMI is calculated by dividing body weight, in kilograms, by height, in meters squared. The child population, however, presents some particularities when dealing with this index, BMI reference values for children and adolescents are defined by percentiles according to age and sex. In this study, the World Health Organization recommendations were followed, and the children were classified as Extreme Thinness (<3rd percentile); Thinness (3rd to 15th percentile); Normal Weight (15th to 84th percentile); Overweight (85th to 97th percentile); and Obesity (>97th percentile) [31].

### Statistical analysis

Initially, to assess patterns of relations among measures and considering that the research was conducted at two different locations, the Spearman correlation test was applied to investigate possible associations between inhibitory control, math performance, and BMI with sex, age, and collection site. Results were considered statistically significant if *p* <0.05 (two-tailed). To establish the effect of each of the variables, hierarchical multiple linear regression analyses were performed to examine the relationship between BMI and inhibitory control with math performance in children. All regression models were adjusted for age, sex, and collection site.

To examine whether the association between inhibitory control and mathematical performance differs in terms of BMI categories we performed a regression analysis between inhibitory control and mathematical performance divided by BMI, adjusted for sex, age, and location. Due to the number of subjects, we divided the BMI into Thinness/Normal Weight and Overweight/Obesity. Moreover, we complemented this analysis by testing the effect of the interaction between BMI and inhibitory control on math performance and performed a moderation analysis using BMI as a moderator, inhibitory control as a predictor, and math performance as an outcome.

Finally, statistical moderation and mediation analyses were tested by using the PROCESS 4.2 macro for SPSS (Version 24.0). A 5000-times bootstrapping procedure was integrated, and asymmetric confidence intervals (CI) were generated (1). We examined if BMI moderates de relationship between inhibitory control (predictor) and math performance (outcome). The approach we used to test moderation is based on ordinary least squares regression analysis for predicting if continuous variables estimate moderate effects.

**Fig 1:**
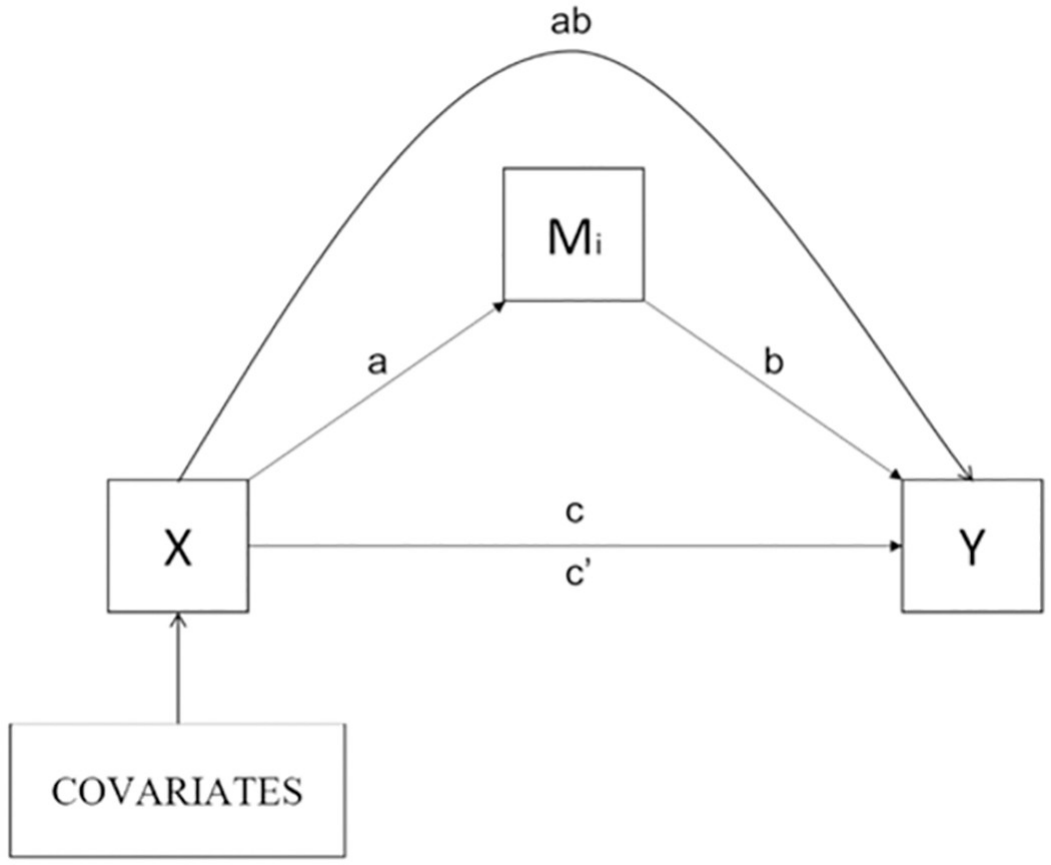
Illustration of the theoretical model of mediation. Where: *a* = effect of *X* on *Mi*; *b* = effect of *Mi* on *Y*; *c* = total effect of *X* on *Y* (when there is no mediation); *ab* = indirect effect (effect of *X* on *Y* passing by *Mi*) and *c’* = direct effect (effect of *X* on *Y* when controlled by indirect effect); *X* = independent variable; *Y* = dependent variable; *Mi* = mediator.

In mediation analyses, we investigate whether inhibitory control statistically mediated (defined as M in the mediation model) the indirect association between BMI and math performance. In this model, BMI was defined as the predictor (X) and math performance as the outcome (Y). To evaluate the power of our model we also tested the inverse regression, considering inhibitory control as outcome and math performance as mediator. The approach we used to test for mediation does not require the presence of a simple association between X and Y to establish indirect effects (Fig 1). To estimate the significance of the indirect effect, we used maximum likelihood linear regression to estimate the coefficients of the mediation model. The indirect effect of X on Y through a mediator (M) is significant if the confidence intervals do not overlap with zero.

Mediation analysis in this cross-sectional study will improve our understanding of indirect relationships between variables and provide suggestive evidence about possible causal relationships [32]. Even when X and Y do not present a direct relationship, the mediation analysis allows the investigation of potential indirect effects mediated by a third variable, since it is plausible that an intermediate variable influences both X and Y, giving rise to an indirect effect [33].

## Results

Demographic parameters, math performance scores, and inhibitory control performance are described in Table 1. In this study, we collected data on a few outcome measures, including Body Mass Index (BMI), math test accuracy, and accuracy in inhibitory control tests on congruent and incongruent trials. It should be noted that for the BMI measure, we had 8 missing data points. It should also be noted that participants with missing data were not included in any analysis involving BMI. Math performance, inhibitory control, and BMI did not differ between sexes.

**Table 1:** Demographic, math performance, and inhibitory control variables by BMI classification. Data are present as average (± standard deviation).

Variable	Thinness and Several Thinness (n = 34)	Normal Weight (n = 72)	Overweight (n = 27)	Obesity (n = 28)
Age (Years)	10.85 (±0.93)	10.76 (±0.85)	10.52 (±0.75)	10.79 (±0.88)
BMI (kg/m^2^)	14.93 (±0.91)	17.65 (±1.29)	20.93 (±0.92)	25.72 (±2.19)
Math Performance (Score)	7.06 (±4.08)	8.67 (±4.43)	8.48 (±5.12)	8.39 (±5.38)
Congruent Accuracy (%)	0.895 (±0.08)	0.89 (±0.09)	0.86 (±0.14)	0.83 (±0.19)
Incongruent Accuracy (%)	0.779 (±0.16)	0.78 (±0.18)	0.75 (±0.17)	0.69 (±0.23)
Congruent Reaction Time (ms)	547.16 (±48.55)	545.77 (±59.66)	526.03 (±67.84)	544.93 (±66.68)
Incongruent Reaction Time (ms)	588.54 (±48.83)	581.69 (±60.26)	565.38 (±76.37)	578.51 (±69.66)

### Association between math performance, inhibitory control, and BMI

To adjust the statistical models of the results described in the following paragraphs, we tested the relationships of age, collection site, and sex with math performance, inhibitory control, and BMI through a correlation analysis. As expected, older ages among children were related to better math performance (ρ = 0.199; p = 0.012). The collection site was correlated with age (ρ = 0.252; p = 0.001), congruent accuracy (ρ = -0.375, p<0.001), and incongruent accuracy (ρ = -0.296, p<0.001). Based on the correlations, the regression and mediation models were adjusted for age, collection site, and sex.

Using Spearman’s correlation analysis, we found that higher BMI was associated with poorer accuracy on the incongruent trials of the Flanker test (ρ = -0.155, p = 0.05). In addition, better math performance was correlated with better inhibitory control (ρ = 0.267, p = 0.001).

**Fig 2:**
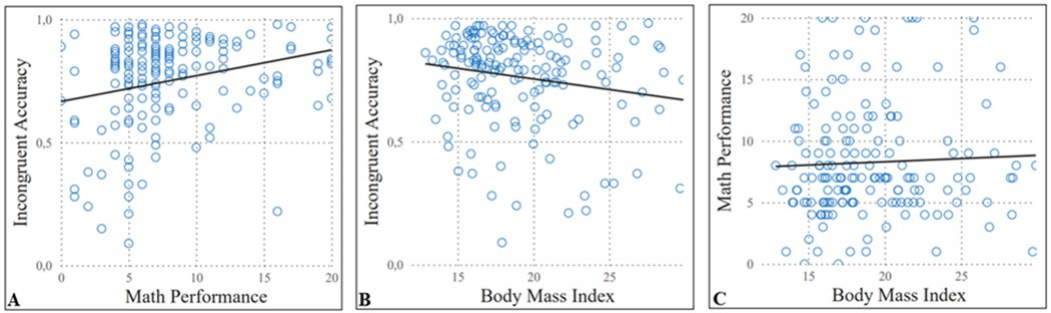
(A) Linear Regression of Inhibitory Control with Math Performance (r^2^ = 0.111, β = 0.267, t = 3.429, p = 0.001), (B) Inhibitory Control with BMI (r^2^ = 0.124, β = -0.220, t = -2.959, p = 0.0001), and (C) no significative relationship of Math Performance with BMI (r^2^ = 0.046, β = 0.042, t = 0.536, p = 0.117).

Hierarchical linear regression analysis reveals that better math performance and lower BMI were associated with greater inhibitory control (Math performance: r^2^ = 0.109, β = 0.274, t = 3.643, p = 0.001; BMI: r^2^ = 0.124, β = -0.220, t = -2.959, p = 0.0001) (Fig 2B). Likewise, greater inhibitory control was associated with better math performance (r^2^ = 0.111, β = 0.267, t = 3.429, p = 0.001) (Fig 2A).

Following, we stratified participants by their BMI (Thinness/Normal (n = 106) and Overweight/Obesity (n = 55). For the kid with Thinness/Normal BMI, the associations between inhibitory control and math performance are best explained by a linear equation (y = 5,27x + 4,01) (r^2^ = 0.043, β = 0.207, t = 2.157, p = 0.033), while for the kids with Overweight/Obesity, this relationship is best explained by an exponential equation (y = 2,17^(1,60x)) (r^2^ = 0.231, β = 0.480, t = 3.988, p< 0.001) (S1 Fig). We found no interaction effect between BMI and inhibitory control on math performance (p = 0.365). Moreover, BMI is not a moderator of the relationship between inhibitory control and mathematical performance (95% CI: -0.35, 1.53).

### Mediation analysis

**Fig 3:**
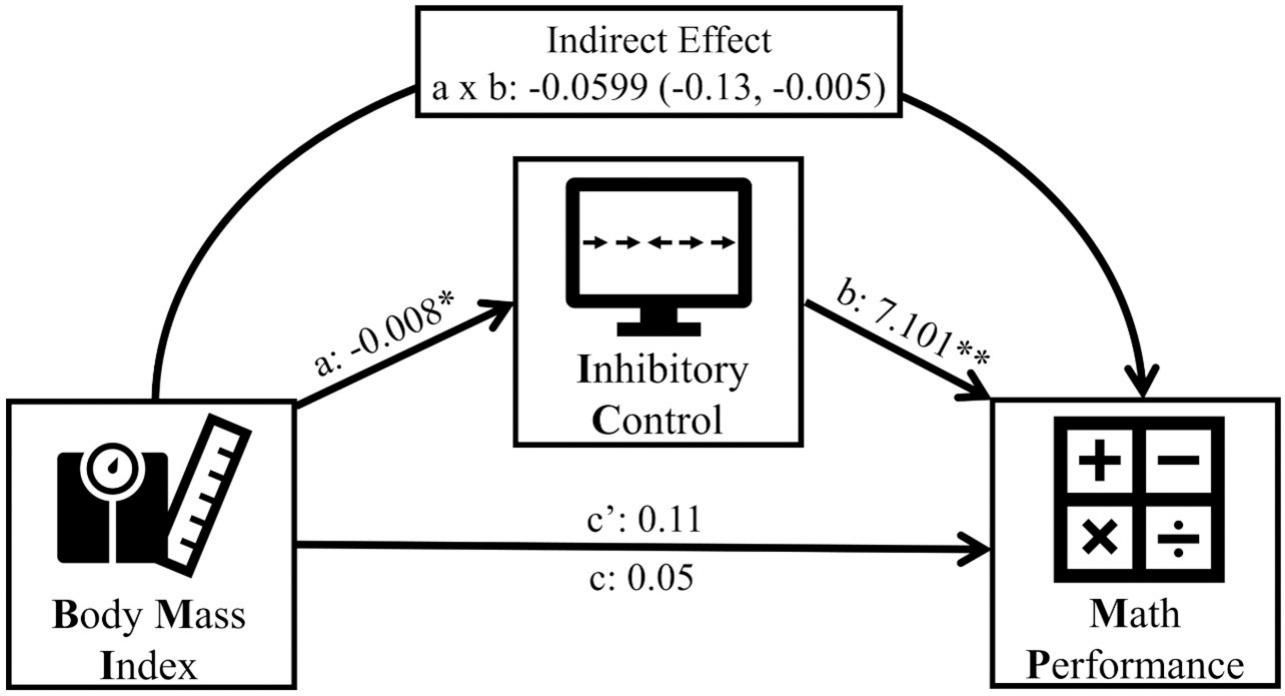
Schematic representation of mediation analysis. a and b represent the non-standardized regression coefficients, c’ the direct effect, c the total effect, and a x b the size of the indirect effect. Data in parentheses are confidence intervals. * p ≤ 0.05; **p ≤ 0.01.

Although there were no direct associations between BMI and math performance (r^2^ = 0.046, β = 0.042, t = 0.536, p = 0.117) (Fig 2C), we tested the hypothesis that inhibitory control constitutes an indirect route (mediator) in the relationship between these two variables. Consistent with our hypothesis, lower BMI was associated with better math performance which was statistically mediated by greater inhibitory control (a: -0.008, p = 0.025; b: 7.10, p = 0.0004; c: 0.05, p = 0.592; c’: 0.11, p = 0.238; Indirect Effect: -0.0599, 95% CI: -0.13, -0.005). This mediation model was adjusted for sex, age, and site (Fig 3). On the other hand, the model that flipped inhibitory control and mathematical performance is not significant (95% CI: -0.0015, 0.0028).

## Discussion

Our specific goals in this study were to examine the relationship between BMI and inhibitory control with math performance in children, to examine whether the association between inhibitory control and mathematical performance differs in terms of BMI categories, and to test whether inhibitory control mediates the relationship between BMI and mathematical performance in children. Our results demonstrate that better inhibitory control is associated with better math performance and lower BMI. Furthermore, the relationship between inhibitory control and math performance varies according to the BMI categories, linearly for children classified with thinness and normal BMI and exponentially for children classified with overweight or obesity. Although we found no direct relationship between BMI and math performance, a higher BMI was indirectly associated with reduced math performance, statistically mediated by performance on the inhibitory control test. This suggests that overweight and obesity in children might impact math performance because of poorer performance in inhibitory control.

Prior studies have demonstrated a relationship between inhibitory control and math performance in children. Faster reaction times, indicative of better performance, during an inhibitory control test were related to better performance on a numerical skills test [34]. Similarly, enhanced inhibitory control has the potential to be a predictive factor for improved mathematical achievement among students in grades 2, 6, and 10. Notably, among various executive functions considered, inhibition consistently featured in all prediction models applied across these grade levels, with the Flanker test (resistance to distractor interference) emerging as the most robust predictor within the grade 6 cohort (ages 10.8 to 12.3 years old) [12]. Our findings align with previous research, corroborating a modest correlation between better inhibitory control and enhanced performance in mathematical addition tasks.

Furthermore, the relationships between inhibitory control and math performance varied according to BMI classification, demonstrating a linear relationship for children with thin/normal BMI, in contrast, an exponential relationship for the kids with overweight/obesity, as well as there are no interactions between BMI with inhibitory control or math performance. This observation holds particular significance as it aligns with the notion that children with higher adiposity or BMI tend to exhibit increased levels of pro-inflammatory cytokines, which have been linked to cognitive and cortical morphometric changes [35, 36]. Moreover, the impact of increased visceral adiposity on cognitive and academic performance appears to be contingent upon BMI status, adding complexity to our understanding [24].

Children with obesity present poorer performance in inhibitory control, in addition to deficits in several other executive functions [10]. More recently, a study involving 3190 children found that higher BMI was associated with lower prefrontal cortex thickness [8], a region of the brain associated with supporting inhibitory control [37]. These findings were further substantiated by another study involving 2,700 children within the same age range (9–11 years old). This study demonstrated that as BMI increased, the thickness of the prefrontal cortex decreased, concomitant with diminished performance on executive function tests. Notably, this study also revealed that changes in cortical thickness mediate the association between BMI and executive functioning [21]. Additionally, a neuroelectric study showed that child with obesity, aged 7 to 9 years, exhibited alterations in P3 and N2 indices, indicative of reduced accuracy and executive control during an inhibition task [9]. These associations may be a biological explanation for our results, that higher BMI and lower efficiency of inhibitory control are moderately associated. Furthermore, the genetic overlap between BMI and executive functions might also explain the association of better executive functions and lower BMI, possible by sharing the same genetic influence [23].

The relationship between BMI and math performance in children has been previously demonstrated. Boys with obesity scored lower on measures of academic performance than overweight or normal weight boys [6]. In another study, this relationship was also found among girls, such that obesity was associated with unsatisfactory academic performance [5]. However, in contrast to this prior literature, we did not find a statistically significant direct relationship between BMI and math performance.

These differences might be explained by the variation in the methods used to assess math performance which. In the present study we assessed math performance through a standardized test independent of the school’s curricular evaluation, while in the studies mentioned above, the curricular assessment itself was used as a parameter of school and math performance. This could have resulted in bias in the assessment method, guidance on the test, and changes in the environment where the test was being performed.

**Fig 4:**
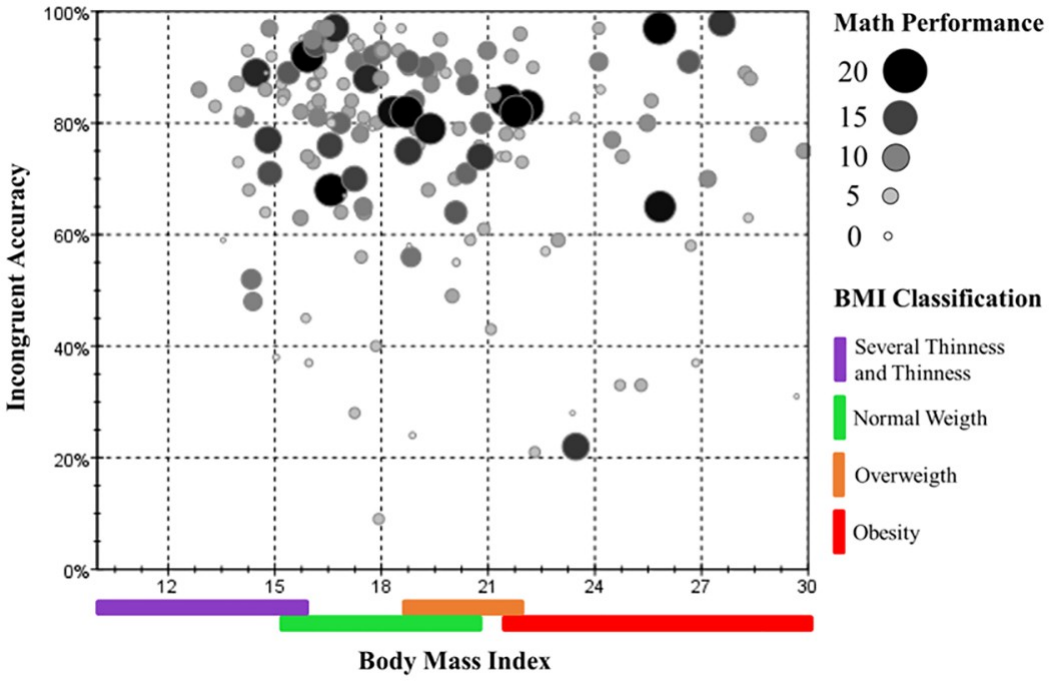
Scatter plot graph. The size and color of the dots represent mathematical performance, and the color legend below the x-axis refers to the BMI classification.

Finally, when plotting math performance, inhibitory control, and BMI in a single graph (Fig 4), we observed that children with better math performance had predominantly normal BMI and performed above 60% of correct answers in the inhibitory control test. Therefore, we investigated the hypothesis that inhibitory control mediates the indirect relationship between BMI and math performance, based on the association of inhibitory control with math performance and with BMI, as well as the absence of a direct relationship between BMI and math performance.

Similar results have already been described for adolescents aged 13.5 to 17 years, where the association between BMI and school performance was mediated by working memory [7], and, in adults, the inhibitory control mediated the indirect relationship between BMI and intelligence [25]. However, despite the numerous studies on BMI, cognition, and brain health in children aged 8 to 13, we believe that we show for the first time that the association between BMI and math performance was statistically mediated by inhibitory control. This provides evidence for a possible mechanism for how overweight and obesity impairs math abilities.

### Limitations

First, our study used BMI as an indirect estimate of body adiposity. Although BMI has known limitations, such as incorrectly classifying highly athletic individuals as obese or overweight [38], in fact we had no highly athletic participants and percentile classification, considering the age and sex of the children was applied since it is considered a valid indicator of body adiposity [39].

In addition, we investigated the relationship between BMI, inhibitory control, and math performance and provided evidence of this relationship by collecting cross-sectional data, limiting the establishment of causal relationships between the variables. Future research should consider the use of longitudinal studies to better understand precedence of effects to establish possible causal pathways.

## Conclusion

In summary, our results showed that there is an association between inhibitory control and performance in mathematics and that this association varies according to BMI. We identified an indirect association between higher body mass indices in children and impaired mathematical performance, through the impact that BMI has on inhibitory control.

## Supporting information

S1 Fig Association between math performance and accuracy in the inhibitory control test for different BMI classifications. (TIF)

S1 Checklist STROBE statement—Checklist of items that should be included in reports of observational studies. (PDF)

S1 File Inclusivity in global research. (DOCX)

## Abbreviations

BMI: Body Mass Index
CI: Confidence Intervals
