# Supplementary figures and images for: Inhibitory control mediates the association between body mass index and math performance in children: A cross-sectional study
Source: PLoS One. 2024 Apr 11;19(4):e0296635. doi: 10.1371/journal.pone.0296635 (PMC11008894; doi:10.1371/journal.pone.0296635)

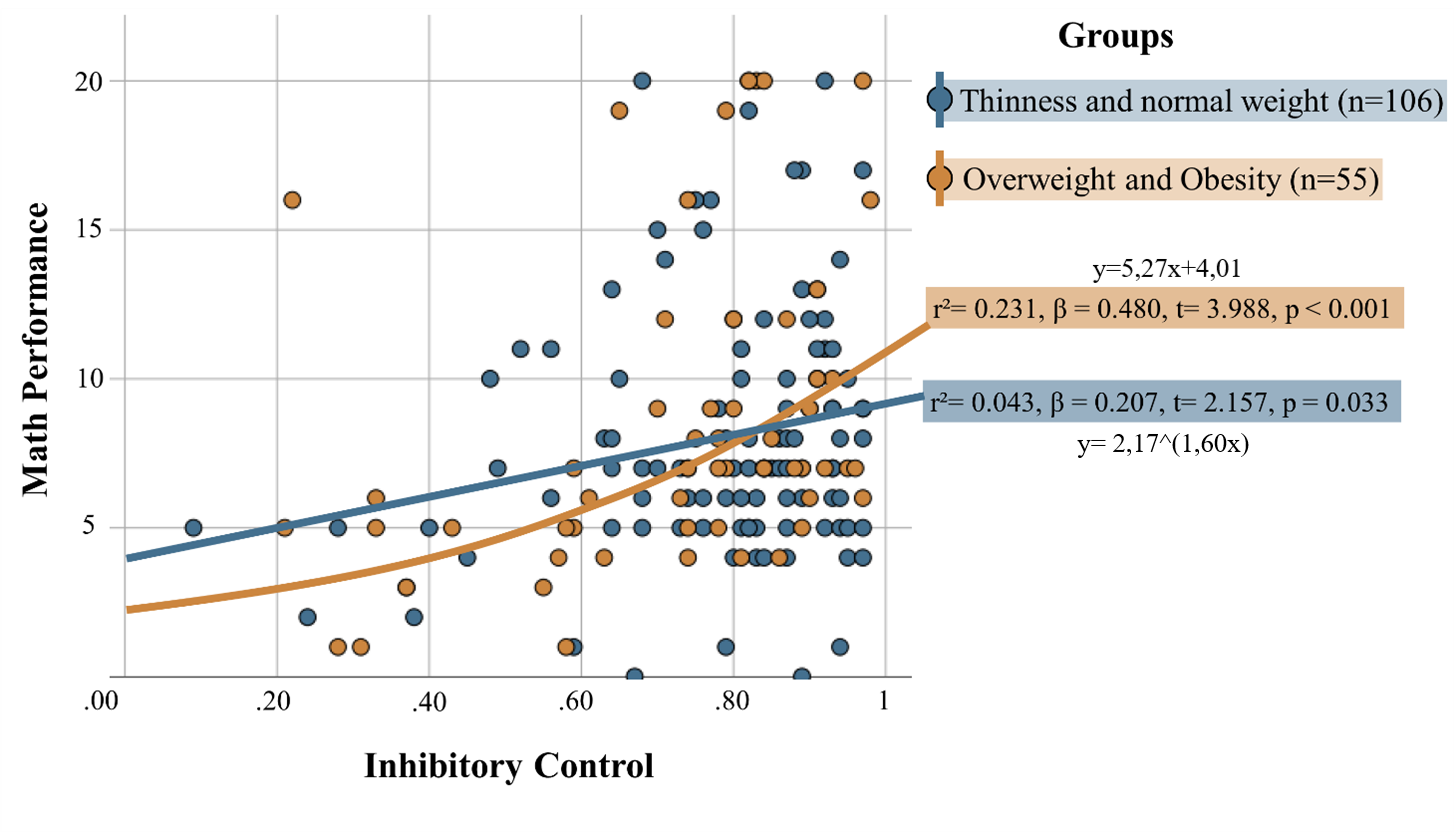

Supplement: S1 Fig — (TIF) [file pone.0296635.s001.tif]
