# Supplementary material for: Inhibitory control mediates the association between body mass index and math performance in children: A cross-sectional study
Source: PLoS One. 2024 Apr 11;19(4):e0296635. doi: 10.1371/journal.pone.0296635 (PMC11008894; doi:10.1371/journal.pone.0296635)
Supplement: S1 Checklist — (PDF) [file pone.0296635.s002.pdf]

STROBE Statement—checklist of items that should be included in reports of observational studies

| | Item No. | Recommendation | Page No. | Relevant text from manuscript |
|---|---|---|---|---|
| **Title and abstract** | 1 | (*a*) Indicate the study's design with a commonly used term in the title or the abstract | 1 | "A cross-sectional study" |
| | | (*b*) Provide in the abstract an informative and balanced summary of what was done and what was found | 2 | |
| **Introduction** | | | | |
| Background/rationale | 2 | Explain the scientific background and rationale for the investigation being reported | 3-4 | |
| Objectives | 3 | State specific objectives, including any prespecified hypotheses | 4 | |
| **Methods** | | | | |
| Study design | 4 | Present key elements of study design early in the paper | 4-5 | |
| Setting | 5 | Describe the setting, locations, and relevant dates, including periods of recruitment, exposure, follow-up, and data collection | 4-5 | "The recruitment period for School 1 was from February to March 2018, and the data collection period was from March to August 2018. The recruitment period for School 2 was from July to August 2019, and the data collection period was from September to December 2019." |
| Participants | 6 | (*a*) *Cohort study*—Give the eligibility criteria, and the sources and methods of selection of participants. Describe methods of follow-up<br>*Case-control study*—Give the eligibility criteria, and the sources and methods of case ascertainment and control selection. Give the rationale for the choice of cases and controls<br>*Cross-sectional study*—Give the eligibility criteria, and the sources and methods of selection of participants | 5 | The inclusion criteria for this study are children between 9 and 13 years of age who do not have any mathematical learning disorders, progressive blindness, deafness, or chronic heart or neurological disease. |
| | | (*b*) *Cohort study*—For matched studies, give matching criteria and number of exposed and unexposed | | |

| | | | | |
|---|---|---|---|---|
| | | *Case-control study*—For matched studies, give matching criteria and the number of controls per case | | |
| Variables | 7 | Clearly define all outcomes, exposures, predictors, potential confounders, and effect modifiers. Give diagnostic criteria, if applicable | 6-8 | |
| Data sources/ measurement | 8* | For each variable of interest, give sources of data and details of methods of assessment (measurement). Describe comparability of assessment methods if there is more than one group | 6-8 | |
| Bias | 9 | Describe any efforts to address potential sources of bias | | |
| Study size | 10 | Explain how the study size was arrived at | 5 | "To ensure that our sample size of 161 was sufficient for detecting these effect sizes, a post-hoc power analysis was conducted using GPower 3.1.9.7 (16) . The results revealed that our sample size had sufficient power between 0.96 and 0.99 when using an alpha of 0.05. Furthermore, a sensitivity power analysis indicated that our sample could detect effect sizes larger than 0.05 with an alpha of 0.05 and a power of 0.8." |

Continued on next page

| | | | | |
|---|---|---|---|---|
| Quantitative variables | 11 | Explain how quantitative variables were handled in the analyses. If applicable, describe which groupings were chosen and why | 6-9 | |
| Statistical methods | 12 | (*a*) Describe all statistical methods, including those used to control for confounding | 7-9 | |
| | | (*b*) Describe any methods used to examine subgroups and interactions | 8=9 | |
| | | (*c*) Explain how missing data were addressed | We have no missing data | |
| | | (*d*) *Cross-sectional study*—If applicable, describe analytical methods taking account of sampling strategy | | |
| | | (*e*) Describe any sensitivity analyses | | |
| **Results** | | | | |
| Participants | 13* | (a) Report numbers of individuals at each stage of study—eg numbers potentially eligible, examined for eligibility, confirmed eligible, included in the study, completing follow-up, and analysed | 9 | A total of 255 children were initially considered eligible for this study. Of these, 174 children were examined for eligibility and 161 were confirmed eligible. All 161 eligible children participated in the study and completed the follow-up period, and their data were included in the analysis. |
| | | (b) Give reasons for non-participation at each stage | | |
| | | (c) Consider use of a flow diagram | | |
| Descriptive data | 14* | (a) Give characteristics of study participants (eg demographic, clinical, social) and information on exposures and potential confounders | 9 | |
| | | (b) Indicate number of participants with missing data for each variable of interest | We have no missing data | |
| Outcome data | 15* | *Cohort study*—Report numbers of outcome events or summary measures over time | | |
| | | *Case-control study*—Report numbers in each exposure category, or summary measures of exposure | | |
| | | *Cross-sectional study*—Report numbers of outcome events or summary measures | 9 | In this study, we collected data on a few outcome measures, including Body Mass Index (BMI), math test accuracy, and accuracy in |

| | | | | |
|---|---|---|---|---|
| | | | | inhibitory control tests on congruent and incongruent trials. |
| Main results | 16 | (*a*) Give unadjusted estimates and, if applicable, confounder-adjusted estimates and their precision (eg, 95% confidence interval). Make clear which confounders were adjusted for and why they were included | 8 | adjusted for sex, age, and location. |
| | | (*b*) Report category boundaries when continuous variables were categorized | 7 | the children were classified as: Extreme Thinness (<3rd percentile); Thinness (3rd to 15th percentile); Normal Weight (15th to 84th percentile); Overweight (85th to 97th percentile); and Obesity (>97th percentile) |
| | | (*c*) If relevant, consider translating estimates of relative risk into absolute risk for a meaningful time period | | Not relevant |

Continued on next page

| | | | | |
|---|---|---|---|---|
| Other analyses | 17 | Report other analyses done—eg analyses of subgroups and interactions, and sensitivity analyses | 10-11 | |
| **Discussion** | | | | |
| Key results | 18 | Summarise key results with reference to study objectives | 11 | Our specific goals in this study were to examine the relationship between BMI and inhibitory control with math performance in children, to examine whether the association between inhibitory control and mathematical performance differs in terms of BMI categories, and to test whether inhibitory control mediates the relationship between BMI and mathematical performance in children. |
| Limitations | 19 | Discuss limitations of the study, taking into account sources of potential bias or imprecision. Discuss both direction and magnitude of any potential bias | 13 | |
| Interpretation | 20 | Give a cautious overall interpretation of results considering objectives, limitations, multiplicity of analyses, results from similar studies, and other relevant evidence | 11-14 | |
| Generalisability | 21 | Discuss the generalisability (external validity) of the study results | | |
| **Other information** | | | | |
| Funding | 22 | Give the source of funding and the role of the funders for the present study and, if applicable, for the original study on which the present article is based | 16 | Not applicable |

*Give information separately for cases and controls in case-control studies and, if applicable, for exposed and unexposed groups in cohort and cross-sectional studies.

**Note:** An Explanation and Elaboration article discusses each checklist item and gives methodological background and published examples of transparent reporting. The STROBE checklist is best used in conjunction with this article (freely available on the Web sites of PLoS Medicine at http://www.plosmedicine.org/, Annals of Internal Medicine at http://www.annals.org/, and Epidemiology at http://www.epidem.com/). Information on the STROBE Initiative is available at www.strobe-statement.org.
